# Complete Genome Sequence of Multidrug-Resistant Edwardsiella ictaluri Strain MS-17-156

**DOI:** 10.1128/genomeA.00477-18

**Published:** 2018-05-31

**Authors:** Hossam Abdelhamed, Hasan C. Tekedar, Ozan Ozdemir, Chuan-Yu Hsu, Mark A. Arick, Attila Karsi, Mark L. Lawrence

**Affiliations:** a College of Veterinary Medicine, Mississippi State University, Mississippi State, Mississippi, USA; b Institute for Genomics, Biocomputing and Biotechnology, Mississippi State University, Mississippi State, Mississippi, USA

## Abstract

Edwardsiella ictaluri is a significant pathogen of cultured fish, particularly channel catfish. Here, we present the complete genome sequence of a multidrug-resistant E. ictaluri strain, MS-17-156, isolated from diseased channel catfish. The genome sequence of this multidrug-resistant strain is expected to help us understand the molecular mechanism of antibiotic resistance in this important pathogen.

## GENOME ANNOUNCEMENT

Edwardsiella ictaluri, a Gram-negative bacillus in the order Enterobacteriales, is the etiological agent of enteric septicemia of catfish (ESC), which is one of the most significant diseases of the commercial catfish industry in the United States (1, 2). Although E. ictaluri is well adapted to channel catfish, it has been linked to mortalities in other fish species, including tilapia, zebrafish, and ayu (3–5). Edwardsiella ictaluri strain MS-17-156 was isolated from a diseased channel catfish in 2017 from the Aquatic Diagnostic Laboratory at the College of Veterinary Medicine, Mississippi State University. E. ictaluri strain MS-17-156 is resistant to tetracycline, oxytetracycline, doxycycline, florfenicol, erythromycin, chloramphenicol, streptomycin, penicillin, novobiocin, azithromycin, and spectinomycin.

Genome sequencing was conducted using HiSeq X Ten (Illumina, San Diego, CA, USA) and MinION (Oxford Nanopore Technologies, Oxford, UK) instruments, producing approximately 441-fold and 36-fold genome coverages, respectively. Illumina reads were trimmed and filtered with Trimmomatic (6), and Nanopore reads were corrected with Canu version 1.6 (7). Contig errors were corrected using Pilon version 1.21 (7). Assembly was performed using MaSuRCA version 3.2.4 (8). The circular physical structure of the chromosome and plasmids was confirmed by PCR. Proteins and noncoding RNAs (ncRNAs) were predicted with the NCBI Prokaryotic Genome Annotation Pipeline (PGAP) (9).

The complete genome sequence ofE. ictaluri strain MS-17-156 consists of one circular chromosome (3,837,027 bp, with an average GC content of 57%) and two circular plasmids named pEI-MS-17-156-1 (135,268 bp, with an average GC content of 52%) and pEI-MS-17-156-2 (4,807 bp, with an average GC content of 51%). The complete genome of E. ictaluri strain MS-17-188 has 99.94% average nucleotide identity (ANI) (10) with the E. ictaluri isolate 93-146 genome (11), and it has 99.41% ANI with the E. ictaluri RUSVM-1 genome (12). A total of 3,837 genes, 3,709 protein-coding sequences (CDS), 273 pseudogenes, and 128 RNA genes (25 rRNAs [9, 8, and 8 for 5S, 16S, and 23S, respectively], 98 tRNAs, and 5 ncRNAs) were predicted in the genome.

pEI-MS-17-156-1 contains resistance genes *floR*, *sul2*, and *tetD*, and this plasmid is nearly identical (almost 100% ANI) to previously sequenced plasmids isolated from a wide variety of Gram-negative bacteria, including Vibrio alginolyticus strain Vb0506 (pVb0506) (13), Klebsiella pneumoniae strain K-109-R plasmid (14), Photobacterium damselae subsp. piscicida (pP91278) (15), Vibrio cholerae strain ICDC-1447 (pVC1447) (16), Salmonella enterica subsp. enterica serovar Senftenberg strain SRC119 (pSRC119-A/C) (17), and Escherichia coli strain PG010208 (pPG010208) (18). An E. ictaluri strain carrying the pEI-MS-17-156-1 plasmid would be potentially resistant to the three antimicrobial drugs (florfenicol, ormetoprim-sulfadimethoxine, and oxytetracycline) that are presently approved for use in aquaculture in the United States. In-depth analysis of this genome is expected to increase our understanding of the mechanisms of antibiotic resistance in E. ictaluri, which would facilitate combating antibiotic resistance and promote antibiotic stewardship programs.

### Accession number(s).

The complete genome sequences of E. ictaluri strain MS-17-156 and its two plasmids have been deposited in GenBank under the accession numbers CP028813 (chromosome), CP028814 (pEI-MS-17-156-1), and CP028815 (pEI-MS-17-156-2).

## References

[1] Hawke JP 1979 A bacterium associated with disease of pond cultured channel catfish, *Ictalurus punctatus*. J Fish Res Bd Can 36:1508–1512. doi:10.1139/f79-219.

[2] Hawke JP 2015 Enteric septicemia of catfish. SRAC publication no. 477. Southern Regional Aquaculture Center, Stoneville, MS.

[3] Soto E, Griffin M, Arauz M, Riofrio A, Martinez A, Cabrejos ME 2012 *Edwardsiella ictaluri* as the causative agent of mortality in cultured Nile tilapia. J Aquat Anim Health 24:81–90. doi:10.1080/08997659.2012.675931. 22838078

[4] Hawke JP, Kent M, Rogge M, Baumgartner W, Wiles J, Shelley J, Savolainen LC, Wagner R, Murray K, Peterson TS 2013 Edwardsiellosis caused by *Edwardsiella ictaluri* in laboratory populations of zebrafish *Danio rerio*. J Aquat Anim Health 25:171–183. doi:10.1080/08997659.2013.782226. 23865817 PMC4077847

[5] Nagai T, Iwamoto E, Sakai T, Arima T, Tensha K, Iida Y, Iida T, Nakai T 2008 Characterization of *Edwardsiella ictaluri* isolated from wild ayu *Plecoglossus altiveli*s in Japan. Fish Pathol 43:158–163. doi:10.3147/jsfp.43.158.

[6] Bolger AM, Lohse M, Usadel B 2014 Trimmomatic: a flexible trimmer for Illumina sequence data. Bioinformatics 30:2114–2120. doi:10.1093/bioinformatics/btu170. 24695404 PMC4103590

[7] Walker BJ, Abeel T, Shea T, Priest M, Abouelliel A, Sakthikumar S, Cuomo CA, Zeng Q, Wortman J, Young SK, Earl AM 2014 Pilon: an integrated tool for comprehensive microbial variant detection and genome assembly improvement. PLoS One 9:e112963. doi:10.1371/journal.pone.0112963. 25409509 PMC4237348

[8] Zimin AV, Marçais G, Puiu D, Roberts M, Salzberg SL, Yorke JA 2013 The MaSuRCA genome assembler. Bioinformatics 29:2669–2677. doi:10.1093/bioinformatics/btt476. 23990416 PMC3799473

[9] Tatusova T, DiCuccio M, Badretdin A, Chetvernin V, Nawrocki EP, Zaslavsky L, Lomsadze A, Pruitt KD, Borodovsky M, Ostell J 2016 NCBI Prokaryotic Genome Annotation Pipeline. Nucleic Acids Res 44:6614–6624. doi:10.1093/nar/gkw569. 27342282 PMC5001611

[10] Yoon S-H, Ha S, Lim J, Kwon S, Chun J 2017 A large-scale evaluation of algorithms to calculate average nucleotide identity. Antonie Van Leeuwenhoek 110:1281–1286. doi:10.1007/s10482-017-0844-4. 28204908

[11] Williams ML, Gillaspy AF, Dyer DW, Thune RL, Waldbieser GC, Schuster SC, Gipson J, Zaitshik J, Landry C, Banes MM, Lawrence ML 2012 Genome sequence of *Edwardsiella ictaluri* 93-146, a strain associated with a natural channel catfish outbreak of enteric septicemia of catfish. J Bacteriol 194:740–741. doi:10.1128/JB.06522-11. 22247535 PMC3264089

[12] Reichley SR, Waldbieser GC, Soto E, Lawrence ML, Griffin MJ 2017 Complete genome sequence of *Edwardsiella ictaluri* isolate RUSVM-1 recovered from Nile tilapia (*Oreochromis niloticus*) in the Western Hemisphere. Genome Announc 5:e00390-17. doi:10.1128/genomeA.00390-17. 28619788 PMC5473257

[13] Li R, Ye L, Zheng Z, Chan EWC, Chen S 2016 Genetic characterization of a *bla*_VEB-2_-carrying plasmid in *Vibrio parahaemolyticus*. Antimicrob Agents Chemother 60:6965–6968. doi:10.1128/AAC.01749-16. 27645248 PMC5075119

[14] Bueno MFC, Francisco GR, de Oliveira Garcia D, Doi Y 2016 Complete sequences of multidrug resistance plasmids bearing *rmtD1* and *rmtD2* 16S rRNA methyltransferase genes. Antimicrob Agents Chemother 60:1928–1931. doi:10.1128/AAC.02562-15. 26729503 PMC4775959

[15] Kim M-J, Hirono I, Kurokawa K, Maki T, Hawke J, Kondo H, Santos MD, Aoki T 2008 Complete DNA sequence and analysis of the transferable multiple-drug resistance plasmids (R plasmids) from *Photobacterium damselae* subsp. *piscicida* isolates collected in Japan and the United States. Antimicrob Agents Chemother 52:606–611. doi:10.1128/AAC.01216-07. 18070959 PMC2224721

[16] Wang R, Liu H, Zhao X, Li J, Wan K 2018 IncA/C plasmids conferring high azithromycin resistance in *Vibrio cholerae*. Int J Antimicrob Agents 51:140–144. doi:10.1016/j.ijantimicag.2017.09.009. 28919196

[17] Harmer CJ, Holt KE, Hall RM 2015 A type 2 a/C2 plasmid carrying the *aacC4* apramycin resistance gene and the *erm*(42) erythromycin resistance gene recovered from two *Salmonella enterica* serovars. J Antimicrob Chemother 70:1021–1025. doi:10.1093/jac/dku489. 25468903

[18] Fernández-Alarcón C, Singer RS, Johnson TJ 2011 Comparative genomics of multidrug resistance-encoding IncA/C plasmids from commensal and pathogenic *Escherichia coli* from multiple animal sources. PLoS One 6:e23415. doi:10.1371/journal.pone.0023415. 21858108 PMC3155540

